# Acute Effects of Static and Proprioceptive Neuromuscular Facilitation Stretching on the Ankle’s Range of Motion and Postural Stability

**DOI:** 10.3390/jfmk11020179

**Published:** 2026-04-29

**Authors:** Rafał Szafraniec, Sebastian Klich, Dawid Koźlenia

**Affiliations:** Faculty of Physical Education and Sport, Wroclaw University of Health and Sport Sciences, 51-612 Wroclaw, Poland; rafal.szafraniec@awf.wroc.pl (R.S.); dawid.kozlenia@awf.wroc.pl (D.K.)

**Keywords:** muscle stretching exercises, PNF stretching, static–passive stretching, joint range of motion, postural balance

## Abstract

**Objectives:** The primary aim of this study was to compare the acute effects of two stretching techniques—static stretching (SST) and proprioceptive neuromuscular facilitation (PNF)—on functional outcomes related to postural balance (stabilographic parameters) and ankle range of motion (ROM; active and passive measures). Furthermore, the study aimed to assess the association between changes in balance- and ROM-related parameters. **Methods:** The study sample consisted of 24 young adults in the age range of 21–24. The SS group (*n* = 12) mean body height was 174.3 ± 7.8 [cm], body weight 68.0 ± 13.1 [kg], and BMI 22.2 ± 2.8 [kg/m^2^]. The PNF group’s (*n* = 12) mean body height was 173.7 ± 7.3 [cm], body weight 68.6 ± 13.5 [kg], and BMI 22.5 ± 3.0 [kg/m^2^]. The subjects performed static stretching or proprioceptive neuromuscular facilitation stretching involving the tibialis anterior, gastrocnemius, and soleus muscles. Before and immediately after the intervention, the active and passive range of plantar and dorsal flexion of the foot and the stability of the body posture in the anterior–posterior plane were measured based on the analysis of the center of pressure (COP) sway. **Results:** The results of the mixed model ANOVA (intervention × time) showed no statistically significant effect of the intervention or interaction between intervention and time for ROM and COP measurements. In both cases, a statistically significant time effect was found. After intervention, significant differences were found in COP variability (*p* = 0.02), COP range (*p* = 0.03), fractal dimension (*p* = 0.04), and sample entropy (*p* = 0.01). Similarly, for range of motion, differences were observed in passive dorsiflexion (*p* < 0.01), active plantarflexion (*p* < 0.01), and passive plantarflexion (*p* = 0.01). Pearson’s correlation analysis did not reveal significant associations between changes in ankle range of motion and COP variables. **Conclusions:** The results of this study indicate that both static and PNF stretching acutely increase the range of motion in the ankle joint; however, they also lead to a decrease in postural stability under more challenging conditions involving visual or vestibular deprivation. The magnitude of the range of motion changes was not associated with alterations in stabilographic parameters.

## 1. Introduction

Stretching exercises are widely utilized in both athletic and rehabilitation settings [1]. Numerous studies have demonstrated that different forms of stretching are commonly incorporated into warm-up and cool-down routines to enhance muscle flexibility [2,3], decrease the risk of musculoskeletal injuries [4,5], and facilitate post-exercise recovery [6,7]. On the other hand, certain forms of stretching used during warm-ups, particularly prolonged static stretching (>60 s), may result in a temporary reduction in strength and power [8]. In clinical practice, stretching interventions are also employed to restore the physiological range of motion (ROM) restricted by soft tissue contractures and to improve postural stability and balance control [9,10]. There are three primary types of stretching: static, dynamic, and proprioceptive neuromuscular facilitation (PNF). In the present study, static and PNF stretching were employed because, despite methodological differences, both techniques allow for comparable durations of muscle elongation.

While the enhancement of both acute and chronic range of motion (ROM) after stretching exercises is well documented [1,2,3], the effect of these exercises on body balance is still uncertain. Only a limited number of studies have investigated the relationship between stretching and balance, typically assessing postural sway in both antero-posterior (AP) and medio-lateral (ML) axes, and their findings are inconsistent [11]. Some research has demonstrated that stretching may enhance postural stability under both dynamic [10,12] and static conditions [13]. Conversely, other studies have reported detrimental effects of stretching on postural control, suggesting that it may temporarily impair balance [14,15]. Moreover, numerous studies have reported no significant alterations in postural stability subsequent to stretching interventions [16,17]. The physiological mechanisms that may explain the effects of stretching on body balance are not yet well documented. Postural balance depends on the integration of multiple components responsible for coordinating sensory input and motor output [17]. Sensory information is derived primarily from the visual and vestibular systems, as well as from proprioceptive input originating from muscle spindles and Golgi tendon organs. The afferent signals provided by these proprioceptors may be modulated by stretch-induced changes in the length and stiffness of the musculoskeletal unit [10], thereby influencing postural control. A second potential mechanism through which stretching may affect balance is an increase in joint range of motion [18]. Lima et al. [19] reported that an increased range of motion may be associated with greater postural sway; however, this effect appears to be transient. To the best of our knowledge, no studies to date have investigated whether the magnitude of stretching-induced changes in ankle dorsiflexion and plantar flexion range of motion affects postural stability specifically in the AP plane, an axis particularly sensitive to ankle proprioception and ROM of dorsiflexion/plantarflexion, which directly may influence lower extremity stretching. In clinical practice, stretching interventions are also applied to restore the physiological ROM restricted by soft tissue contractures and to improve postural stability and balance control. Validated clinical tools like the Cumberland Ankle Instability Tool (CAIT) enable reliable identification of at-risk athletes and monitoring of intervention efficacy in sports settings [20]. Chronic ankle instability is not only a consequence of trauma, but rather a complex condition involving neuromuscular deficits and alterations in postural control. From the clinical and sport perspective, these sensorimotor alterations predispose individuals to ongoing functional limitations and injury risk, underscoring the need for targeted interventions addressing both joint range of motion and dynamic balance. Recently, Hertel (2002) presented chronic ankle instability as a sensorimotor disorder featuring neuromuscular control deficits and proprioceptive impairments [21]. Moreover, McKeon et al. (2008) demonstrated spatiotemporal postural control deficits during single-leg stance [22], while Wikstrom et al. (2010) linked delayed peroneal activation to recurrent sprain risk in athletes [23]. These sensorimotor alterations justify targeted interventions addressing both ROM and dynamic stability [24]. Therefore, the primary aim of this study was to compare the acute effects of two stretching techniques—static stretching and proprioceptive neuromuscular facilitation (PNF)—on functional outcomes related to postural balance (stabilographic parameters) and ankle range of motion (ROM; active and passive measures). Furthermore, the study aimed to assess the association between changes in balance- and ROM-related parameters in simple connections and multivariate patterns to determine whether they reflect a common construct or represent separate functional dimensions.

## 2. Materials and Methods

### 2.1. Study Design

The study was designed to investigate the acute effects of two types of stretching on postural control and ankle joint range of motion. It was a single-blind randomized controlled trial. Participants were randomly assigned to one of two intervention groups: static stretching (SST) or proprioceptive neuromuscular facilitation stretching (PNF). Group allocation was determined using a simple computer-generated randomization procedure performed with the online tool Randomizer.org, which generated a 1:1 allocation sequence for the SST and PNF groups. The investigator responsible for outcome assessment remained blinded to group allocation throughout the testing procedure. Before the intervention, active and passive ankle joint ranges of motion were measured. Participants were then familiarized with the postural stability measurement procedure through one non-recorded trial per stance condition on the force platform. Two stance conditions were assessed: (1) quiet standing with head in a neutral position (HNP) aligned in the Frankfort horizontal plane, and (2) standing with head tilted backward (HTB). For each stance condition, one recorded trial was conducted as the pre-test. Following the baseline measurements, participants performed either static stretching training (SST) or proprioceptive neuromuscular facilitation (PNF) stretching according to their group allocation. Immediately after the intervention ended, the second postural stability assessment (post-test) was conducted under the same conditions as the pre-test. Finally, active and passive ankle joint ranges of motion were re-measured to assess changes following the stretching intervention. All participants underwent the same testing sequence, and both pre- and post-intervention assessments were performed under identical, standardized measurement conditions. The measurements were taken between 9:00–11:00 a.m.

### 2.2. Participants

An a priori power analysis was conducted using G*Power software v. 3.1.9.7 [25] to estimate the minimum sample size required to detect a within–between interaction in a repeated-measures design with two groups (static stretching vs. PNF) and two measurement points (pre- and post-intervention). Assuming an effect size of f = 0.35 [26], an alpha level of 0.05, and statistical power of 0.80, the analysis indicated that a minimum total sample of 20 participants was required. Therefore, the final sample of 24 participants was considered sufficient to detect the main effects of time and group, as well as the time × group interaction.

The study sample consisted of 24 young adults aged 21–24, recruited through advertisements posted on the university website and social media platforms. The participants were recreationally active and had no evident orthopedic and/or neurological pathologies, balance disorders, or lower-limb muscular or joint injuries within the previous six months. All participants were familiarized with the study procedure and voluntarily signed an informed consent to participate. The study was approved by the Research Ethics Committee of Wroclaw University of Health and Sport Sciences (16/2018) and conducted in line with the Declaration of Helsinki guidelines. The SST group (*n* = 12) had a mean body height of 174.3 ± 7.8 cm, body weight of 68.0 ± 13.1 kg, and BMI of 22.2 ± 2.8 kg/m^2^. The PNF group (*n* = 12) had a mean body height of 173.7 ± 7.3 cm, body weight of 68.6 ± 13.5 kg, and BMI of 22.5 ± 3.0 kg/m^2^. The descriptive characteristics of the two groups indicated comparable baseline anthropometric profiles prior to the intervention. In addition, before testing, the participants were instructed to follow the examination procedures and to avoid behaviours that could acutely affect the measurements. In particular, they were asked to refrain from vigorous physical activity 48 h before the session and to maintain their usual dietary and hydration routine. They were also instructed to avoid medications, stimulants, or other substances that could influence range of motion or postural stability on the day of testing unless medically necessary. Because of the acute nature of the study, all participants were assessed within the same experimental session using an identical measurement sequence.

### 2.3. Measurements

#### 2.3.1. Anthropometric Measurements

Stature was measured using a calibrated anthropometer (Swiss Anthropometer, GPM Anthropological Instruments, DKSH Ltd., Zürich, Switzerland), and body mass was determined with a bioelectrical impedance analyzer (InBody230, InBody Co., Ltd., Cerritos, CA, USA) [11]. Both height and weight measurements were obtained with an accuracy of 0.1 cm and 0.1 kg, respectively. The body mass index (BMI) was calculated using the formula: body mass [kg]/height^2^ [m^2^]. The measurements were taken according to the protocol outlined by the International Society for the Advancement of Kinanthropometry (ISAK) [27].

#### 2.3.2. Range of Motion Measurements

Active (AROM) and passive (PROM) ranges of motion for dorsiflexion (DF) and plantar flexion (PF) were assessed using a standard manual goniometer by an experienced examiner. Measurements were performed with the knee flexed at 10° to simulate a weight-bearing position. The endpoint was defined as first firm tissue resistance without compensatory pelvic tilting or lumbar lordosis. Intrarater ICCs for manual goniometer measurements were 0.95–0.97 for plantar flexion and 0.85–0.96 for dorsiflexion. During the assessment, participants were positioned supine on an examination table with a thin pillow placed beneath the knee to maintain slight flexion (~10°). The axis of rotation of the goniometer was aligned with the lateral malleolus, while the fixed arm was positioned parallel to the lateral midline of the fibula, and the movable arm was aligned with the lateral midline of the fifth metatarsal bone. Points corresponding to the goniometer placement were marked on the skin using a black indelible marker. Each measurement was performed three times, and the mean value of the three trials was used for further analysis.

#### 2.3.3. Postural Stability

Postural control was evaluated using a force platform (Kistler 9281CA, Winterthur, Switzerland). Participants from both groups completed two stabilographic assessments: the first conducted before stretching exercises (pre-test) and the second immediately afterward (post-test). Each assessment consisted of two 30-s trials of quiet bipedal stance performed with eyes closed: one with the head maintained in a neutral position (HNP), aligned with the Frankfort horizontal plane, and the other with the head tilted backward (HTB), representing a more challenging postural condition due to altered vestibular input [28]. The order of trials was counterbalanced across participants.

Foot placement was standardized, with heels positioned 5 cm apart, to ensure consistency across trials and participants. All tests were performed barefoot, with subjects instructed to stand comfortably yet as still as possible, arms relaxed alongside the body. A one-minute rest interval was provided between trials to minimize potential fatigue effects. Data were collected at a sampling frequency of 100 Hz. Following a “ready” signal from the participant, the examiner initiated the recording, which began 10 s later to eliminate transient adaptation effects. Ground reaction forces were processed to compute the center-of-pressure (COP) trajectories.

Postural performance was evaluated in the anterior–posterior (AP) plane using sway variability and range, with lower values of these parameters reflecting superior postural stability [29]. Postural control strategies were characterized using the fractal dimension and sample entropy of the COP signal, which quantify the complexity and irregularity of postural sway, respectively. Higher COP complexity values are interpreted as greater adaptability of the postural control system to varying sensory or environmental demands [30]. Regarding the COP entropy, higher values denote increased irregularity of postural sway, reflecting a more automated, efficient, and less attentionally demanding mode of postural control regulation [31]. In contrast, lower entropy values are indicative of more regular and predictable sway dynamics, suggesting greater conscious involvement in balance maintenance and, consequently, a diminished degree of postural automaticity [32].

### 2.4. Stretching Protocols

All participants in the study were familiar with stretching techniques.

#### 2.4.1. PNF Stretching

- Gastrocnemius Muscle

Participants were positioned supine with the knee joint fully extended. The examiner grasped the participant’s foot and passively dorsiflexed the ankle to first firm tissue resistance without pain or compensatory movements. In this position, the examiner applied manual resistance to the plantar surface of the foot while the participant performed a moderate submaximal isometric plantarflexion contraction (comfortable effort without pain). The isometric contraction was sustained for 10 s, followed by a 10-s relaxation period. The examiner then further increased dorsiflexion to renewed firm tissue resistance (new stretching endpoint), and this position was maintained for 30 s. One complete cycle consisted of this hold–relax sequence (10-s contraction + 10-s relaxation + 30-s stretch). A second isometric contraction was performed within the same stretched position, followed by the same relaxation and stretch procedure. Three complete cycles were performed on one limb, after which the procedure was repeated on the contralateral limb [9,33].

- Soleus Muscle

The stretching procedure for the soleus muscle followed the same sequence as described above. Participants were positioned prone with the knee flexed to 90°, allowing isolation of the soleus muscle. The examiner grasped the foot and performed passive dorsiflexion of the ankle, followed by the same series of isometric contractions and passive stretches as in the gastrocnemius protocol.

- Tibialis Anterior Muscle

For the tibialis anterior muscle, participants were positioned supine with the knee joint extended. The examiner grasped the participant’s foot and performed passive plantarflexion of the ankle joint to induce muscle stretching. The protocol of isometric contraction, relaxation, and subsequent passive stretching was identical to that applied in the gastrocnemius muscle procedure.

#### 2.4.2. Static Stretching

Static stretching was performed by the examiner in the same positions used during the PNF stretching. Each stretch was applied passively until the participant reported a sensation of mild stretching discomfort and was maintained for 30 s. The procedure was repeated three times for each lower limb.

### 2.5. Statistical Analysis

All statistical analyses were performed using Statistica 13.0 (StatSoft Inc., Tulsa, OK, USA). The alpha level was set at *p* = 0.05. The Shapiro–Wilk test was applied to verify the normality of data distribution. Descriptive statistics were calculated for each variable, including mean values, standard deviations (SD), and 95% confidence intervals (CI). For within-group differences (pre–post), Cohen’s *d* values were computed as the mean change divided by the pooled standard deviation of pre- and post-test scores. The magnitude of the effect was interpreted according to conventional thresholds: small (*d* = 0.2), moderate (*d* = 0.5), and large (*d* = 0.8) [34]. To assess the effects of the experimental conditions over time, a mixed-model ANOVA with one between-subject factor (Condition: PNF vs. static stretching) and one within-subject factor (time: PRE vs. POST) was performed. When significant main effects or interactions were detected, appropriate post hoc comparisons with adjustment for multiple testing were conducted. Effect sizes (partial η^2^) were also calculated to evaluate the magnitude of observed effects. Further, the simple association between changes in analyzed parameters calculated as the difference between post- and pre-measurements was assessed with r-Pearson correlation. A hierarchical cluster analysis was conducted to identify similarities among the investigated variables. All variables were standardized using z-scores to eliminate the influence of differences in measurement scales and variances. Standardization was performed according to the formula: z = (value − mean)/standard deviation. As a result, each variable was rescaled to have a mean of 0 and a standard deviation of 1. This procedure ensured that all parameters contributed equally to the analysis, preventing variables with larger numerical ranges from disproportionately affecting the outcomes of the cluster and factor analyses. The Euclidean distance was used as the measure of dissimilarity, and Ward’s method was applied as the linkage criterion. This approach minimizes within-cluster variance and typically produces more homogeneous and interpretable clusters compared to other agglomeration techniques.

## 3. Results

Table 1 presents the descriptive statistics of balance parameters and range of motion measured before and after the intervention, with results shown separately according to group allocation, considering the type of intervention. A non-statistically significant interaction effect was found between condition * time (*p* > 0.05). However, a significant main effect was found for time (*p* < 0.05).

For balance measurements obtained during quiet standing with the head in a neutral position, no significant condition × time interactions or group effects were observed (*p* > 0.05). As shown in Table 1, the pre–post changes were small in both groups, with trivial-to-small effect sizes across all variables. For the head tilted backward condition, no significant condition × time interactions or group effects were found for any stabilographic parameter (Table 2). However, significant main effects of time were observed for COP variability (*p* = 0.02), COP range (*p* = 0.03), fractal dimension (*p* = 0.04), and sample entropy (*p* = 0.01) (Table 2). As shown in Table 1, the changes were generally greater in the PNF group than in the static stretching group, particularly for COP variability (Δ = 1.28 vs. 0.47 mm), COP range (Δ = 5.03 vs. 3.89 mm), and sample entropy (Δ = −0.23 vs. −0.04). Regarding ankle range of motion, no significant condition × time interactions or group effects were observed (Table 2). However, significant main effects of time were found for passive dorsiflexion (*p* < 0.01), active plantarflexion (*p* < 0.01), and passive plantarflexion (*p* = 0.01) (Table 2). As shown in Table 1, both groups increased ROM after the intervention, with numerically greater changes in the PNF group for passive dorsiflexion (Δ = 1.96° vs. 1.46°), active plantarflexion (Δ = 4.58° vs. 2.38°), and passive plantarflexion (Δ = 4.58° vs. 1.46°).

Pearson’s correlation analysis revealed no statistically significant relationship between ROM variables and stabilographic parameters (*p* > 0.05) (Table 3). Only for active plantarflexion, and the fractal dimension of the COP path was a moderate but non-significant trend observed (r = 0.34; *p* = 0.087). The absence of simple linear relationships prompted further analyses aimed at exploring multidimensional dependency patterns.

**Figure 1:**
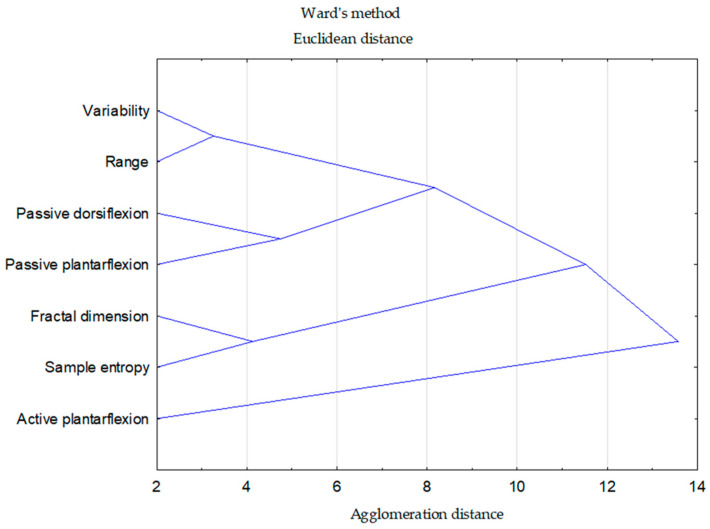
Cluster analysis results for changes in balance parameters and range of motion.

The dendrogram obtained with Ward’s method revealed several distinct clusters (Figure 1). The strongest association was observed between COP variability and range, which merged at the smallest linkage distance, indicating a high degree of similarity. Similarly, fractal dimension and sample entropy formed another compact subcluster. A separate pair of highly related variables was identified for the passive measures: passive dorsiflexion and passive plantarflexion. At a higher level of the hierarchy, these clusters were grouped, suggesting partial convergence between active and passive measures. The most distinct variable within the dataset was active plantarflexion, which only merged with the other clusters at the highest linkage distance, highlighting its unique profile relative to the remaining parameters.

## 4. Discussion

In summary, the present study demonstrated that both static and PNF stretching acutely increased ankle ROM, specifically passive dorsiflexion, active plantarflexion, and passive plantarflexion. However, in this sample of healthy young adults, these acute changes in ankle ROM were not accompanied by detectable changes in the selected postural stability parameters. These findings suggest that short-term increases in ankle mobility may not necessarily translate into measurable changes in postural control under the conditions assessed in the present study [35,36].

The results indicated that a single bout of stretching, regardless of the method applied (static stretching or PNF), did not induce differential effects on postural control in young adults. No significant group × time interactions were observed, indicating that both stretching protocols produced a comparable acute response pattern. Thus, any observed changes were more strongly related to the measurement time point than to the specific stretching method used. In the quiet standing condition, no significant alterations in balance parameters were noted. However, in the more challenging stance with head extension, significant time effects were observed in COP variability, range, fractal dimension, and sample entropy. These findings suggest that stretching may transiently affect postural control when the task requires greater sensory integration and balance demands [37,38]. Additionally, significant time effects were detected in ankle range of motion, specifically in passive dorsiflexion, active plantarflexion, and passive plantarflexion. These outcomes confirm the expected immediate improvement in joint mobility following both static and PNF stretching. Such acute changes in ROM may be related to transient reductions in passive resistance and increased stretch tolerance following the stretching intervention [39,40]. The cluster analysis provided an additional perspective on the relationships among the analyzed variables. COP variability and range, as well as fractal dimension and sample entropy, clustered together, which is consistent with the fact that these parameters describe related aspects of postural sway. Passive dorsiflexion and passive plantarflexion also formed a separate pairing, likely reflecting their common ROM-related character. At the same time, these groupings did not indicate a consistent common structure linking ROM changes with changes in postural stability, which is in line with the lack of significant correlations observed in the present study. These observations are in line with previous studies that showed similarities between static and PNF stretching in improving joint ROM, while evidence on immediate effects on postural control remains limited, with some gaps in current knowledge [41].

The observed dissociation between increased ankle mobility and the absence of clear balance improvements indicates that postural control depends on mechanisms extending beyond peripheral flexibility alone, including sensory integration and central regulation processes [23,42]. Moreover, mechanical adaptations reduce passive muscle stiffness and passive resistive torque, facilitating greater joint excursion [43,44]. According to Podczarska-Głowacka et al. [45], balance adaptations may necessitate prolonged, task-specific neuromuscular engagement beyond isolated joint flexibility improvements. Therefore, acute static and PNF stretching effectively increase ankle mobility; however, this alone may be insufficient to induce measurable improvements in postural stability. Postural control requires the integration of vestibular, proprioceptive, and visual information, and may therefore be less sensitive to acute flexibility gains than to the sensory demands of the task itself [39].

Taken together, the results demonstrate that in young adults, both static and PNF stretching acutely enhance ankle joint range of motion but do not differentially influence balance. Instead, subtle changes in postural control appear mainly under more demanding conditions, indicating that the acute effects of stretching on balance may be context-dependent rather than method-dependent. The findings obtained in the head-tilted-backward condition further support the view that any acute effects of stretching on postural regulation are more likely to become evident when postural demands are increased and the contribution of vestibular and proprioceptive mechanisms is more strongly challenged [3]. From a practical perspective, the present findings suggest that acute static and PNF stretching may be useful when the immediate goal is to increase ankle ROM, but such interventions alone should not be expected to produce measurable improvements in postural stability in healthy young adults. If the aim is to influence balance, stretching may need to be combined with more specific balance-oriented exercises or training strategies. Considering potential limitations, this study focused on young healthy adults, which may limit generalizability to older or clinical populations. Due to the nature of stretching interventions, participant blinding was not feasible, which represents a potential source of performance bias. However, the assessor-blind design and standardized protocols minimized detection bias in ROM and postural stability measurements. A small sample size (per group) may suggest comparable acute effects rather than definitive equivalence between SST and PNF, warranting confirmation in larger trials. In addition, the relatively small sample size may have limited the ability to detect subtle between-group differences, particularly for condition × time interactions. Because the participants were healthy young adults, ceiling effects for some balance outcomes also cannot be excluded. The acute design does not allow inferences about long-term adaptations or the interaction of repeated stretching and balance training. Finally, the correlation and cluster analyses were exploratory and should be interpreted cautiously, especially in light of the absence of significant correlations. Future research should explore combined intervention protocols, neurophysiological correlates of post-stretching balance changes, and integration with functional performance outcome measures.

## 5. Conclusions

The results of this study indicate that both static and PNF stretching acutely increase ankle range of motion, yet these improvements do not translate directly into enhanced postural balance. The magnitude of ROM changes was not associated with alterations in stabilographic parameters, suggesting that acute improvements in ankle flexibility were not accompanied by measurable changes in balance in this sample and under the conditions applied. These conclusions apply to young healthy adults considered as a combined group and should not be extrapolated to sex-specific populations or to clinical and older cohorts.

## Figures and Tables

**Table 1:** Descriptive statistics of postural parameters and the ankle range of motion.

Variable	PNF Stretching				Static Stretching			
	Mean ± SD (95%CI)				Mean ± SD (95%CI)			
	Pre	Post	Δ	ES	Pre	Post	Δ	ES
	Head in neutral position							
Variability (mm)	4.08 ± 1.48 (3.14–5.02)	3.8 ± 1.04 (3.14–4.46)	−0.29 ± 1.48 (−1.23–0.66)	0.22	4.56 ± 1.61 (3.54–5.58)	4.7 ± 1.15 (3.97–5.43)	0.14 ± 1.81 (−1.01–1.29)	0.10
Range (mm)	19.34 ± 6.8 (15.02–23.66)	20.66 ± 7.14 (16.12–25.19)	1.31 ± 9.13 (−4.49–7.11)	0.19	21.98 ± 6.35 (17.94–26.02)	23.96 ± 6.61 (19.77–28.16)	1.98 ± 5.14 (−1.28–5.25)	0.30
Fractal dimension (-)	1.41 ± 0.06 (1.37–1.44)	1.4 ± 0.07 (1.36–1.45)	0 ± 0.06 (−0.04–0.03)	0.14	1.39 ± 0.09 (1.34–1.45)	1.42 ± 0.06 (1.38–1.45)	0.03 ± 0.09 (−0.03–0.08)	0.37
Sample entropy (-)	1.13 ± 0.28 (0.95–1.31)	1.07 ± 0.35 (0.84–1.29)	−0.06 ± 0.37 (−0.3–0.17)	0.19	0.96 ± 0.37 (0.73–1.2)	0.97 ± 0.21 (0.83–1.1)	0 ± 0.42 (−0.26–0.27)	0.03
	Head tilted backward							
Variability (mm)	4.91 ± 1.18 (4.16–5.65)	6.19 ± 1.65 (5.14–7.24)	1.28 ± 1.2 (0.52–2.05)	0.87	4.89 ± 1.94 (3.66–6.12)	5.37 ± 1.48 (4.43–6.3)	0.47 ± 1.99 (−0.79–1.74)	0.27
Range (mm)	23.94 ± 4.24 (21.25–26.64)	28.97 ± 6.25 (25–32.94)	5.03 ± 4.76 (2–8.05)	0.89	23.16 ± 7.78 (18.22–28.1)	27.05 ± 7.66 (22.19–31.92)	3.89 ± 11.93 (−3.69–11.48)	0.51
Fractal dimension (-)	1.43 ± 0.06 (1.39–1.47)	1.39 ± 0.06 (1.35–1.43)	−0.04 ± 0.06 (−0.08–0)	0.67	1.44 ± 0.06 (1.4–1.48)	1.43 ± 0.07 (1.39–1.47)	−0.01 ± 0.04 (−0.04–0.02)	0.15
Sample entropy (-)	1.01 ± 0.2 (0.89–1.14)	0.79 ± 0.25 (0.63–0.94)	−0.23 ± 0.23 (−0.37–−0.08)	0.99	1.03 ± 0.29 (0.85–1.21)	0.99 ± 0.27 (0.82–1.16)	−0.04 ± 0.27 (−0.21–0.13)	0.14
	Range of motion							
Active dorsiflexion (°)	12.04 ± 4.94 (8.9–15.18)	12.58 ± 3.53 (10.34–14.82)	0.54 ± 3.78 (−1.86–2.94)	0.12	9.33 ± 2.84 (7.53–11.14)	11.38 ± 4.36 (8.61–14.14)	2.04 ± 2.62 (0.38–3.7)	0.56
passive dorsiflexion (°)	15.25 ± 4.75 (12.23–18.27)	17.21 ± 4.73 (14.21–20.21)	1.96 ± 2.5 (0.37–3.55)	0.41	13.83 ± 3.36 (11.7–15.97)	15.29 ± 4.45 (12.46–18.12)	1.46 ± 2.35 (−0.03–2.95)	0.36
active plantarflexion (°)	56.58 ± 8.3 (51.31–61.86)	61.17 ± 7.45 (56.43–65.9)	4.58 ± 4.96 (1.43–7.74)	0.57	61 ± 5.37 (57.59–64.41)	63.38 ± 5.07 (60.15–66.6)	2.38 ± 5.83 (−1.33–6.08)	0.45
passive plantarflexion (°)	61.71 ± 8.65 (56.21–67.21)	66.29 ± 7.17 (61.74–70.84)	4.58 ± 5.74 (0.94–8.23)	0.56	67.67 ± 4.61 (64.74–70.6)	69.13 ± 5.74 (65.48–72.77)	1.46 ± 4.28 (−1.26–4.18)	0.28

**Table 2:** Repeated-measures ANOVA results for postural parameters in the head tilted backward condition and the ankle range of motion.

Variable	Effects	F	*p*	Partial η^2^
Variability (mm)	Group	0.57	0.46	0.03
	Time	6.84	0.02 *	0.24
	Interaction	1.46	0.24	0.06
Range (mm)	Group	0.47	0.50	0.02
	Time	5.79	0.03 *	0.21
	Interaction	0.09	0.76	0.01
Fractal dimension (-)	Group	1.11	0.30	0.05
	Time	4.39	0.04 *	0.17
	Interaction	1.45	0.24	0.06
Sample entropy (-)	Group	1.45	0.24	0.06
	Time	6.97	0.01 *	0.24
	Interaction	3.33	0.08	0.13
Passive dorsiflexion (°)	Group	0.95	0.34	0.04
	Time	11.9	<0.01 *	0.35
	Interaction	0.25	0.62	0.01
Active plantarflexion (°)	Group	1.76	0.20	0.07
	Time	9.92	<0.01 *	0.31
	Interaction	1.00	0.33	0.04
Passive plantarflexion (°)	Group	2.99	0.10	0.12
	Time	8.56	0.01 *	0.28
	Interaction	2.29	0.14	0.09

* statistically significant results *p* < 0.05. Group refers to the between-subject factor (stretching condition: static stretching vs. PNF), Time refers to the within-subject factor (pre- vs. post-intervention), and Interaction represents the Group × Time effect. F and *p* values are derived from two-way repeated-measures ANOVA. Partial η^2^ indicates the effect size of each factor, reflecting the proportion of variance explained after accounting for other effects in the model.

**Table 3:** Pearson correlation results of changes in balance parameters and range of motion.

Variable	Passive Dorsiflexion	Active Plantarflexion	Passive Plantarflexion
Variability	r = 0.21	r = 0.13	r = 0.23
	*p* = 0.28	*p* = 0.52	*p* = 0.25
Range	r = 0.21	r = 0.12	r = 0.13
	*p* = 0.29	*p* = 0.54	*p* = 0.50
Fractal dimension	r = 0.074	r = 0.342	r = 0.28
	*p* = 0.72	*p* = 0.09	*p* = 0.15
Sample entropy	r = −0.17	r = 0.21	r = −0.01
	*p* = 0.39	*p* = 0.30	*p* = 0.99

## Data Availability

The data presented in this study are available on request from the corresponding author. The data are not publicly available due to privacy concerns.
